# Effects of Aging on Cerebral Oxygenation during Working-Memory Performance: A Functional Near-Infrared Spectroscopy Study

**DOI:** 10.1371/journal.pone.0046210

**Published:** 2012-09-28

**Authors:** Anouk Vermeij, Arenda H. E. A. van Beek, Marcel G. M. Olde Rikkert, Jurgen A. H. R. Claassen, Roy P. C. Kessels

**Affiliations:** 1 Donders Institute for Brain, Cognition and Behaviour, Radboud University Nijmegen, Nijmegen, The Netherlands; 2 Alzheimer Centre Nijmegen, Department of Geriatric Medicine, Radboud University Nijmegen Medical Centre, Nijmegen, The Netherlands; 3 Department of Medical Psychology, Radboud University Nijmegen Medical Centre, Nijmegen, The Netherlands; Charité University Medicine Berlin, Germany

## Abstract

Working memory is sensitive to aging-related decline. Evidence exists that aging is accompanied by a reorganization of the working-memory circuitry, but the underlying neurocognitive mechanisms are unclear. In this study, we examined aging-related changes in prefrontal activation during working-memory performance using functional Near-Infrared Spectroscopy (fNIRS), a noninvasive neuroimaging technique. Seventeen healthy young (21–32 years) and 17 healthy older adults (64–81 years) performed a verbal working-memory task (n-back). Oxygenated and deoxygenated hemoglobin concentration changes were registered by two fNIRS channels located over the left and right prefrontal cortex. Increased working-memory load resulted in worse performance compared to the control condition in older adults, but not in young participants. In both young and older adults, prefrontal activation increased with rising working-memory load. Young adults showed slight right-hemispheric dominance at low levels of working-memory load, while no hemispheric differences were apparent in older adults. Analysis of the time-activation curve during the high working-memory load condition revealed a continuous increase of the hemodynamic response in the young. In contrast to that, a quadratic pattern of activation was found in the older participants. Based on these results it could be hypothesized that young adults were better able to keep the prefrontal cortex recruited over a prolonged period of time. To conclude, already at low levels of working-memory load do older adults recruit both hemispheres, possibly in an attempt to compensate for the observed aging-related decline in performance. Also, our study shows that aging effects on the time course of the hemodynamic response must be taken into account in the interpretation of the results of neuroimaging studies that rely on blood oxygen levels, such as fMRI.

## Introduction

Working memory is one of the cognitive functions that is particularly sensitive to aging-related decline [1]–[3]. Aging is associated with both decrements in working-memory capacity and alterations in working-memory processing networks [4]. In contrast to young adults, older people have been reported to demonstrate more bilaterally organized prefrontal components of the working-memory circuitry [5]. Further, reduced hippocampal activity [6] and additional activation of prefrontal regions during working-memory performance has been reported [7].

The precise underlying neurocognitive mechanisms of altered prefrontal activation in older adults are unclear. Two main hypothesis regarding the alterations have been proposed: the dedifferentiation and compensation hypotheses. The dedifferentiation view holds that due to decreased neural responsivity and increased neural noise, the cortical representations become less distinctive in the aging brain. This leads to the recruitment of similar brain systems by different neurocognitive functions, regardless of whether it is beneficial for behavioral performance or not [8]. According to the compensatory reorganization hypothesis, additional recruitment of brain regions may represent compensatory mechanisms recruited to counteract aging-related neurocognitive decline, in order to achieve or attempt to achieve the same performance levels as younger adults [9], [10].

The recruitment of additional neural circuitry is not unique to the aging brain. The Compensation-Related Utilization of Neural Circuits Hypothesis (CRUNCH) proposes that, irrespective of age, neural engagement varies with the level of task demand [9]. According to CRUNCH, declined neural efficiency in older adults leads to recruitment of more neural resources than young adults at low levels of task demand. However, as task demands increase, older adults reach a limit of neural resource availability, resulting in underactivation relative to young adults at higher loads. Consistent with this notion, previous functional Magnetic Resonance Imaging (fMRI) and electroencephalography (EEG) research showed that working-memory processing is modulated by working-memory load and age [11]–[15].

The current study utilizes functional Near-Infrared Spectroscopy (fNIRS), a noninvasive neuroimaging technique, to gain more insight into aging-related changes in functional prefrontal activation patterns during working-memory performance. The principles of fNIRS have been extensively described (for review see [16]–[18]). fNIRS is particularly sensitive to the microvasculature [19], [20] and enables monitoring of concentration changes in cortical oxygenated ([O_2_Hb]) and deoxygenated hemoglobin ([HHb]) with high temporal resolution. Based on the tight coupling of neural activity and oxygen delivery [21], both increases in [O_2_Hb] and decreases in [HHb] are taken as indicator of cortical activation [22]. fNIRS has been used to gain more insight into the physiological mechanisms of the BOLD response during fMRI [22] and may, for example, be a useful technique for brain-computer interfaces [23]. In comparison to fMRI, fNIRS has low running costs, a high portability, it is relatively insensitive to movement artifacts, and it enables measurements in a natural setting. These advantages of fNIRS make the technique perfectly suitable to study functional brain activation in a broad range of participants including children, psychiatric patients, and elderly.

Few studies have applied fNIRS in the field of cognitive aging. These fNIRS studies showed an aging-related decline in prefrontal activity during performance of an arithmetic task [24], a verbal fluency task [25], a Stroop task [26], three subtests of the Wechsler Adult Intelligence Scale [27], or a walking-while-talking task [28]. Herrmann et al. [29] found aging-related decline in prefrontal activity during a verbal-fluency task. Young adults showed left-hemispheric lateralization, but older adults did not show a lateralization effect. Tsjuii et al. [30] reported that the right inferior frontal cortex was more activated than the left inferior frontal cortex in young adults during performance of a deductive reasoning task. Older adults additionally recruited the left inferior frontal cortex in order to compensate for age-related decline. To our knowledge, no fNIRS studies on the relationship between prefrontal activation and working-memory load in older adults have been published to date.

In fMRI research, the n-back paradigm has reliably and validly been employed in establishing cerebral activity patterns in the prefrontal cortex in relation to increasing working-memory load [31], [32]. Performance of the n-back task requires on-line monitoring, updating, and manipulation of remembered information. Therefore it is assumed that large demands are placed on several key processes within working memory. The executive processing components of working-memory are assumed to be more affected by aging than the storage components [33]. In contrast to, for example, delayed-matching-to-sample tasks, the n-back task requires not only passive maintenance, but also executive processing operations, especially at higher task loads. Therefore, the n-back task is highly suitable for cognitive aging research.

fMRI studies reported increased prefrontal activity in older adults in comparison with young adults during performance of a verbal 1-back task. Higher working-memory loads (2- and 3-back) resulted in reduced prefrontal activity [11], [12]. Nagel et al. [13] reported that in young adults, activation increased linearly from 1-back up to the highest level of working-memory load (3-back) in several prefrontal areas except the right ventrolateral cortex. By contrast, for older adults no linear increase was reported, except for the left frontopolar cortex, suggesting compensatory activation at low load. The EEG study of Missonnier et al. [14] showed that in older adults the amplitude of the frontal early positive-negative working memory (PNwm) component, reflecting neural activity, reached maximal values during the less demanding 0-back task. In young adults, maximal values were reached during performance of the 2- and 3-back task.

The aim of the current study was to investigate how prefrontal brain activity is changing as a function of parametric manipulations of working-memory load in both young and older adults. Based on theories of prefrontal compensatory mechanisms in aging [9], [34] we hypothesized that, compared to young, older people show increased prefrontal recruitment during working-memory performance. To test this hypothesis we measured prefrontal activation during performance of a verbal n-back task using fNIRS.

## Methods

### Participants

Thirty-four healthy Dutch speaking volunteers participated in the study (17 young adults, 10 female, mean age = 25.9±3.0 years, range 21–32; 17 older adults, 11 female, mean age = 70.7±5.2 years, range 64–81). The young adults were recruited from the social network of the authors and were just as naive about the study as the older adults. The older adults were recruited from local bridge, chess, and senior clubs. They did not experience memory problems (self report), were living independently at home, and had unimpaired cognitive function as assessed with the Mini Mental State Examination [35] (mean score = 29.2±0.9, range 27–30). The educational level of the participants was assessed based on the Dutch educational system [36], using seven categories (1 = less than primary school, 7 = university degree). The educational level slightly differed between the young (M = 6.7±0.7, range 5–7) and older adults (M = 5.5±0.9, range 4–7) (Mann Whitney U = 49.50, p<.001), but all participants completed secondary school or higher. Also, in older adults, educational level often underestimates actual intelligence, because many had limited access to advanced schooling. All participants had an IQ >85 as estimated by the Dutch version of the National Adult Reading Test [37]. All were right-handed and had normal or corrected-to-normal vision. None of the participants had a history of neurological/psychiatric disease or used psychopharmacological drugs. Six older adults used antihypertensive medication. All participants refrained from alcohol, caffeine, and nicotine from at least 3 hours before the experimental session.

### Ethics statement

The research proposal of the present study was submitted to the regional medical-ethics committee (CMO Arnhem-Nijmegen, no. 2009/198), but was deemed exempt from formal medical ethical evaluation, because the study does not fall within the remit of the Medical Research Involving Human Subjects Act (WMO). All participants gave written informed consent. All data were anonymized before analysis. The study was performed according to the Helsinki Declaration.

### Experimental paradigm

**Figure 1:**
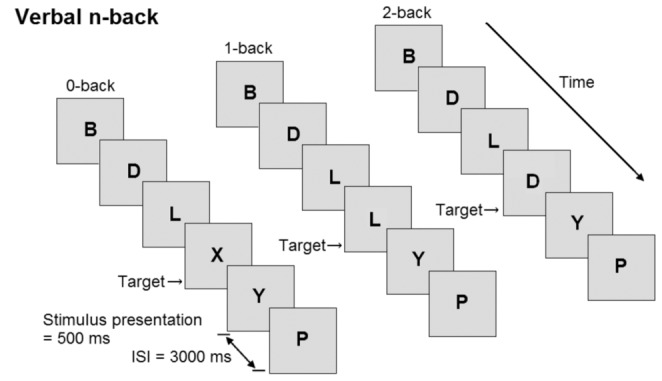
Schematic overview of the verbal n-back task. Stimulus presentation was 500 ms, interstimulus interval (ISI) 3000 ms. During the ISI, a fixation cross was displayed. Participants were allowed to respond until the next stimulus appeared.

Participants performed three versions of a verbal n-back task (see Figure 1): 0-back (control condition), 1-back (low working-memory load condition), and 2-back (high working-memory load condition). Prior to all conditions, participants practiced the task for one minute and received feedback about their performance. The conditions were preceded by a baseline period of one minute, during which a black fixation cross was displayed at the center of the 15 inch screen. Stimuli were presented in black on a grey background using E-prime 2.0 software (Psychology Software Tools, PA, USA), which also registered the behavioral performance. Stimulus presentation time was 500 ms. Interstimulus interval was set to 3000 ms to optimize the behavioral response of the older participants [33], [38]. All conditions consisted of 60 trials of which 17 were target trials. The distribution of targets over the task period was random. In each trial participants indicated whether the stimulus was a target by pressing the button under the right index finger, or a non-target by pressing the button under the right middle finger (PST Serial Response Box, Psychology Software Tools Inc., PA, USA). On each trial, a letter that was randomly selected from a set of 20 consonants was displayed at the center of the screen. In the 0-back condition, the letter “X” was defined as target. In the 1-back and 2-back condition, the target was any letter that was identical to the letter presented n trials before, while the letter “X” was no longer shown. The whole experimental procedure lasted around 20 minutes per participant. To minimize effects of fatigue, participants were able to rest a couple of minutes between conditions.

### Instrumentation

[O_2_Hb] and [HHb] (µmol/L) were measured by a continuous-wave NIRS device (Oxymon, Artinis Medical Systems™, The Netherlands) using light of three wavelengths (775, 845, 904 nm). Near-infrared light penetrates biological tissue such as the skull and brain rather easily. In the cerebral cortex, the near-infrared light is absorbed by the chromophores O_2_Hb and HHb, which have different absorption spectra. Assuming constant scattering [39] and by using the modified Lambert-Beer Law, it is possible to calculate the concentration of these chromophores in the penetrated brain tissue based on changes in the detected light intensity. Both increases in [O_2_Hb] and decreases in [HHb] are indicators of cortical activation. Concentration changes in total hemoglobin ([tHb]), defined as the sum of changes in [O_2_Hb] and [HHb], are used as an indicator of alterations in total blood volume. Since absolute concentration of the chromophores O_2_Hb and HHb cannot be determined by a continuous-wave NIRS device, all measurements are expressed as absolute concentration changes from an arbitrary zero at the start of the measurement period. Data were sampled at 125 Hz.

**Figure 2:**
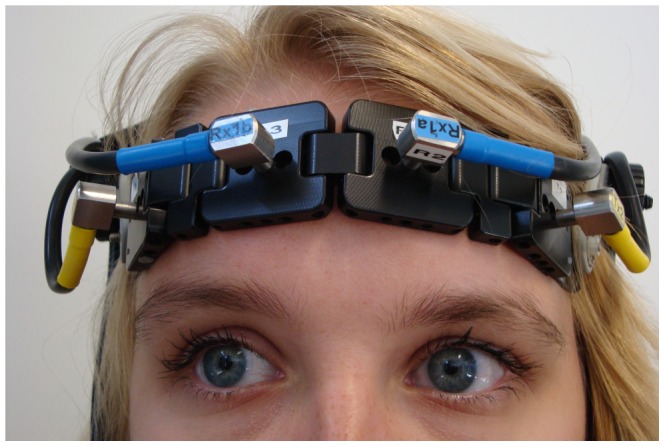
Positioning of the two pairs of fNIRS optodes. The subject of the photograph has given written informed consent (as outlined in the PLoS consent form) to publication of her image.

Two pairs of optodes were bilaterally attached to the forehead and were tightly fixed in a customized headband (Spencer technologies™, Seattle, Wa). The detection optodes were placed 25–30 mm above the midpoint of the eyebrow, at approximately FP1 and FP2 according to the international 10–20 electrode system (see Figure 2). The emission optodes were laterally placed at approximately F7 and F8. The emitter-detector spacing was 50 mm to minimize contamination from the extra-cerebral circulation and maximize signal intensity [40], [41]. Based on Monte Carlo simulation, the average photon path from emitter to detector is estimated to be ellipsoid or banana-shaped with a penetration depth of approximately 2 to 3 cm [42], [43]. Hence, the cerebral areas under investigation were the left and right superior and middle frontal gyrus (Brodmann's area 10/46) [44]. The differential pathlength factor (DPF), which accounts for the increased distance travelled by light due to scattering, is age-dependent [45]. For the young adults, DPF was calculated by the formula 4.99+0.067×Age^0.814^. At present however, no data are available on the actual variation of DPF in adults aged above 50 years. Therefore, in the older adults, the DPF was set to 6.61, corresponding to age 50 [45]. Although this results in inaccurate estimation of true DPF in the elderly, it will have no effect on the accuracy of our assessment of relative changes in [O_2_Hb] and [HHb] within and between conditions [46].

Cerebral hemodynamic changes during the n-back task may potentially be influenced by systemic changes. To account for this, blood pressure was measured simultaneously using a photoplethysmography cuff on the index or middle finger of the left hand of the participant (Finometer, Finapres Medical Systems™, the Netherlands). Blood pressure data were available from 15 young and 14 older adults.

### Data processing

fNIRS data were analyzed using commercially available software (Oxysoft, Artinis Medical Systems™, The Netherlands). A moving average window of 1 s was applied to the [O_2_Hb] and [HHb] signals to filter out the noise of the heart beat frequency. The first three trials (all non-targets) of all conditions were excluded from behavioral and fNIRS data analyses. For the fNIRS signals, the value at the start of the fourth trial was taken as zero. Changes of [O_2_Hb], [HHb], and [tHb] were recalculated for 180 s from this point. Subsequently, each task period was divided into six time segments of 30 s. Mean values of [O_2_Hb], [HHb], and [tHb] were calculated for each time segment. To quantify asymmetric prefrontal activation, the laterality index (LI) was calculated for the time segments, and for each task the average LI was determined. LI was defined as: 100×(|ΔQ_left_|−|ΔQ_right_|)/(|ΔQ_left_|+|ΔQ_right_|), where Q_left_ and Q_right_ represent the fNIRS parameters [O_2_Hb], [HHb], and [tHb], measured by the left and right fNIRS channel respectively. The value of the LI ranges from −100, indicating pure right-hemispheric dominance, to +100, indicating pure left-hemispheric dominance. A value near zero indicates no dominant hemisphere.

### Statistical analysis

Statistical analysis was performed using PASW Statistics software version 18.0 (SPSS Inc., Chicago, IL, USA). Behavioral performance on the verbal n-back tasks was assessed by number of hits, misses, correct rejections, and false alarms. The non-parametric discrimination index (i.e., sensitivity) A′ was calculated by the formula: 0.5+((hit rate−false alarm rate)×(1+hit rate−false alarm rate))/(4×hit rate×(1−false alarm rate)). A′ is a performance variable derived from signal detection theory [47] and ranges from 0.5 (chance level) to 1 (perfect discrimination between targets and non-targets). Shapiro-Wilk tests and Q-Q plots indicated that the assumptions for performing an ANOVA were not met. Accuracy (i.e. A′) of the young and older adults was compared by Mann-Whitney U tests. In both groups, the effects of working-memory load were established by performing Wilcoxon signed-rank tests. For reaction time on targets and non-targets a 2 (group: young, old)×3 (load: 0-,1-,2-back) repeated measures ANOVA was performed.

The assumption of normality was met by the fNIRS data acquired during the verbal n-back task, as was established by Shapiro-Wilk tests. For each of the hemodynamic measures, [O_2_Hb], [HHb], and [tHb], a 2 (location: left, right hemisphere)×2 (group: young, old)×3 (load: 0-,1-,2-back)×6 (time: time segments 1, 2, 3, 4, 5, 6) repeated measures ANOVA was performed. Due to violations of the sphericity assumption, Greenhouse-Geisser corrections were applied. Significant main and interaction effects were further analyzed by means of planned contrasts. With respect to the aim of the current study, primarily significant effects involving the factors load and age are reported, as these are the factors of interest. Because we expected that activation in the left and right hemisphere is differentially affected by the factors load and age, data from the left and right channel were analyzed separately. With respect to the factor time, a trend analysis was performed to characterize the time course over the whole task period.

## Results

### Behavioral performance

Table 1 shows the behavioral results of the young and older adults during the n-back tasks. Compared to the control condition, both low and high working-memory load led to a declined accuracy in older adults (0- vs. 1-back z = −3.045, p<.001; 0- vs. 2-back z = −3.295, p<.001; 1- vs. 2-back z = −2.480, p = .011), but not in young adults. However, with greater load reaction times on targets and non-targets increased significantly in both groups (p<.001). No group×load interaction was found for reaction time.

**Table 1:** Accuracy and reaction times (Mean ± SD) for the verbal n-back tasks.

		Young adults			Older adults			U- or F-statistic
**A′ (accuracy)**	0-back	0.99	±	0.01	0.99	±	0.01	139.00
	1-back	0.98	±	0.02	0.97	±	0.03	94.00
	2-back	0.99	±	0.01	0.94	±	0.05	51.50**
**RT target (ms)**	0-back	496.60	±	79.92	660.95	±	125.05	20.85**
	1-back	550.56	±	77.49	689.89	±	99.91	20.64**
	2-back	643.03	±	134.01	870.59	±	154.52	21.04**
**RT non-target (ms)**	0-back	498.38	±	84.32	610.38	±	87.41	14.46**
	1-back	573.76	±	101.29	667.98	±	97.97	7.60*
	2-back	728.73	±	155.20	832.19	±	159.75	3.67

For group comparisons (young vs. older adults) of accuracy Mann-Whitney-U test values are given. For comparisons of reaction time F values are given.

** p≤.001,

* p<.01.

### fNIRS results

**Figure 3:**
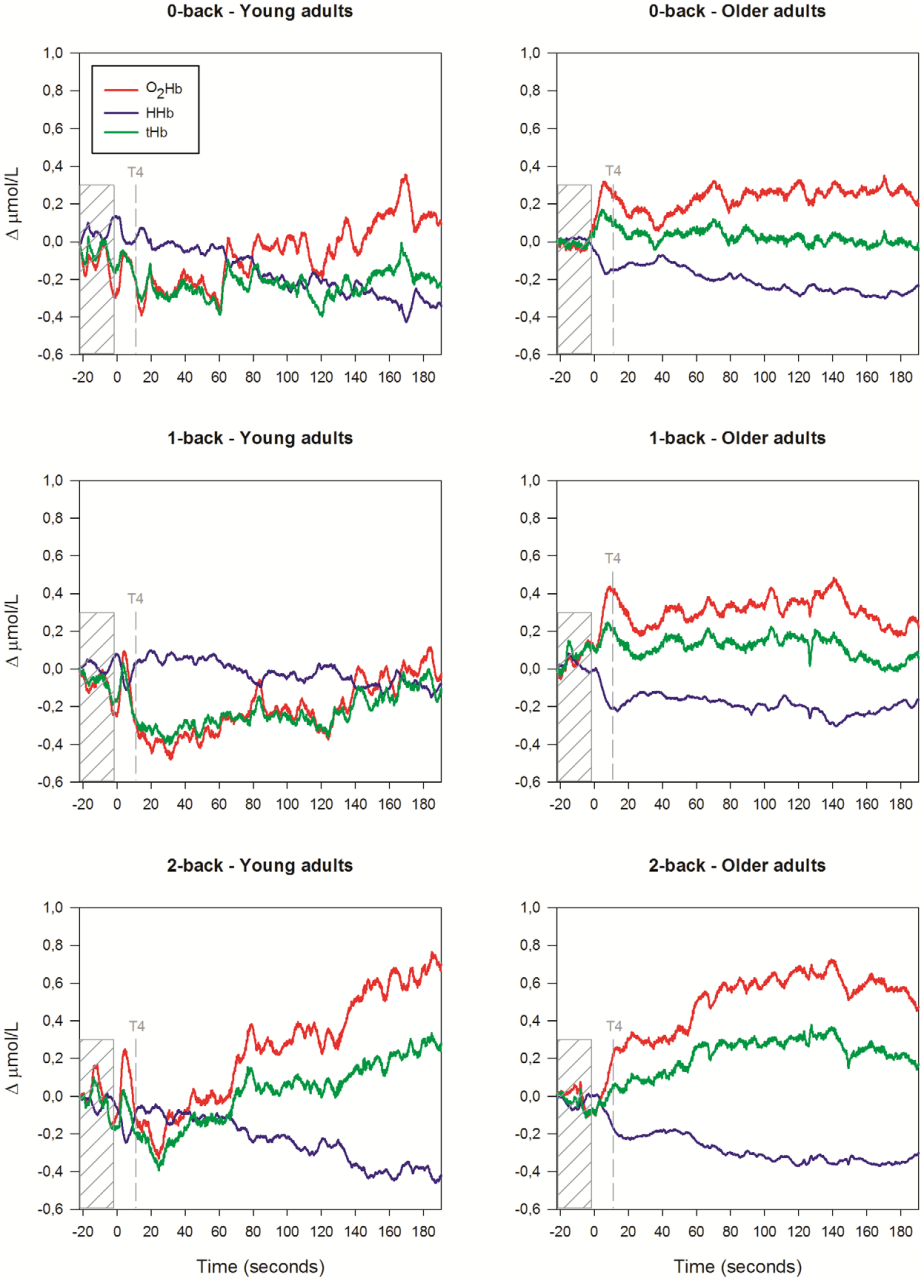
Grand average waveforms of [O_2_Hb], [HHb] and [tHb] changes in the prefrontal cortex. The Figure shows raw fNIRS signals of young and older adults during performance of the verbal 0-back task (*upper panels*), 1-back task (*middle panels*) and 2-back task (*lower panels*). The last 20 s of the pre-task baseline period are marked in the Figure. The n-back task starts at 0 s. The first three trials were excluded from further data analysis. The start of the fourth trial has been marked by the dashed line (T4). For illustrative purposes, in this Figure the signals were nulled at −22 s and were averaged over the left and right optode pair.

**Figure 4:**
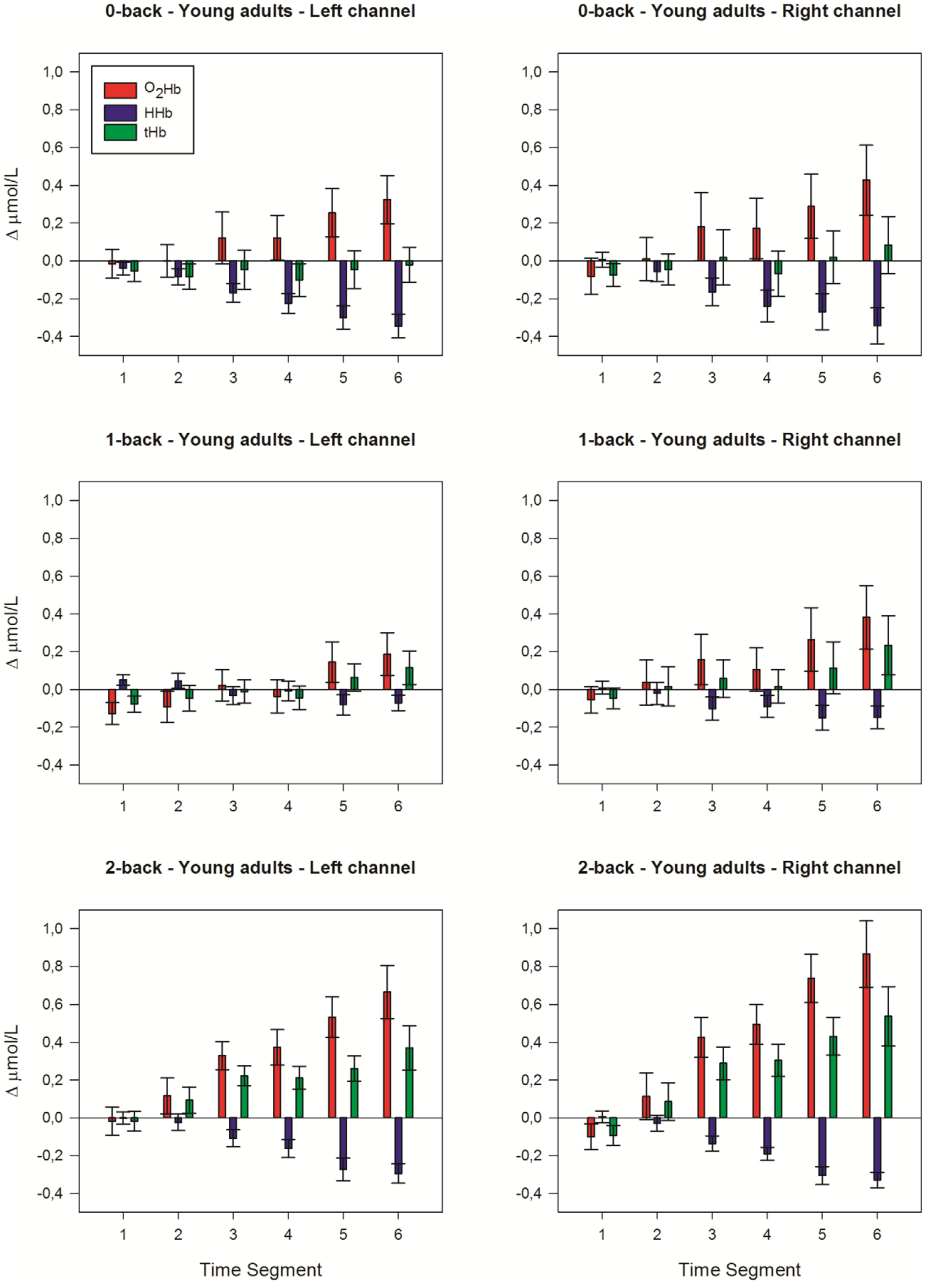
Hemodynamic concentration changes in young adults. Mean (± SEM) changes of [O_2_Hb], [HHb] and [tHb] in the left and right hemisphere during the six 30-s time segments of the verbal 0-back task (*upper panels*), 1-back task (*middle panels*) and 2-back task (*lower panels*).

**Figure 5:**
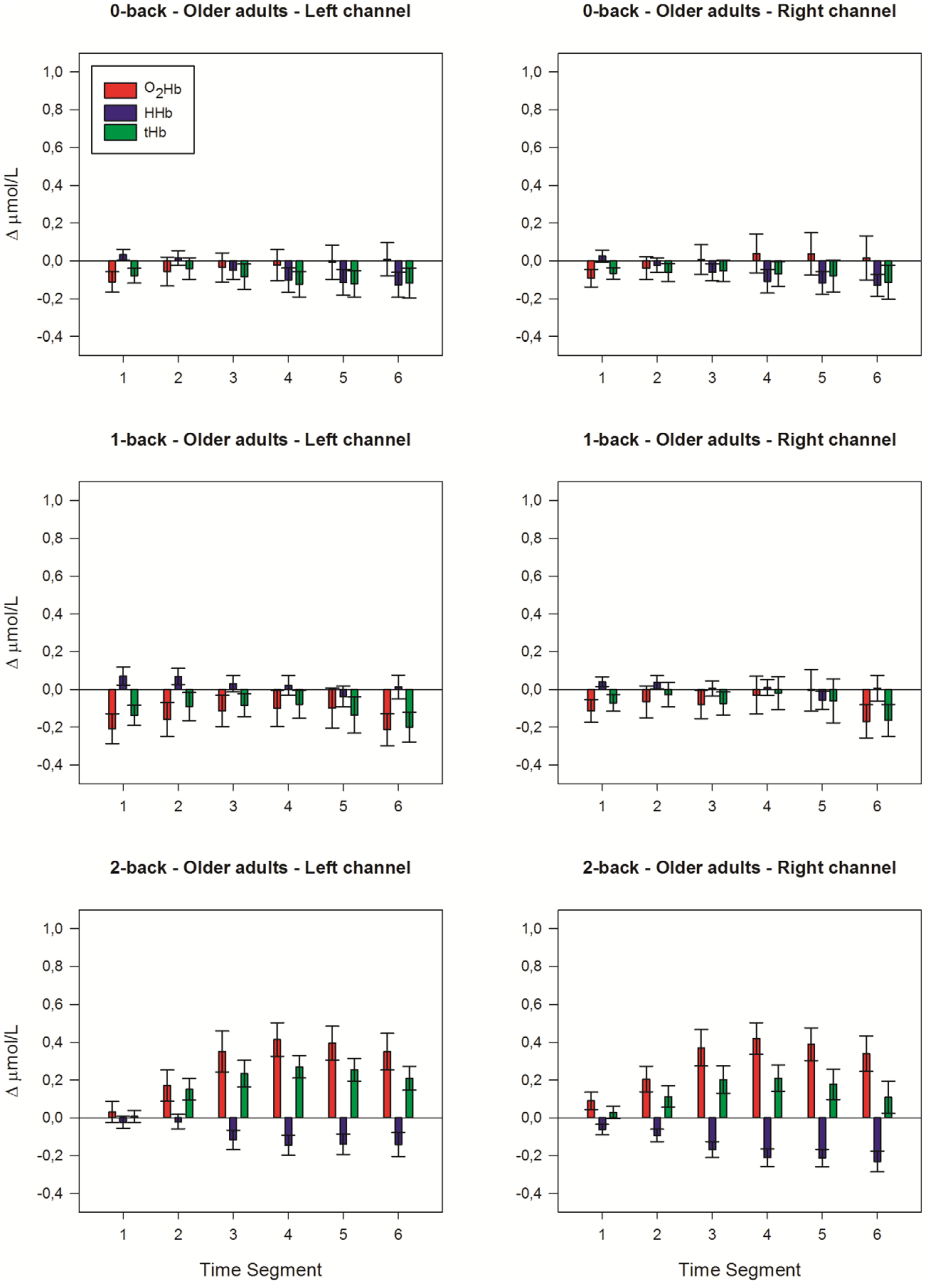
Hemodynamic concentration changes in older adults. Mean (± SEM) changes of [O_2_Hb], [HHb] and [tHb] in the left and right hemisphere over the six 30-s time segments of the verbal 0-back task (*upper panels*), 1-back task (*middle panels*) and 2-back task (*lower panels*).

Figure 3 shows the courses over time of the raw fNIRS signals that were measured during the 20 s pre-task baseline period and during the 180 s verbal n-back tasks. Figures 4 and 5 display the mean changes of [O_2_Hb], [HHb], and [tHb] for each of the six 30 s time segments in which we divided the 180 s n-back tasks. The four-way interaction of location×time×group×load was not significant ([O_2_Hb]: F_(5.23, 167.50)_ = 1.70, p = .134; [HHb]: F_(5.55, 177.68)_ = 0.44, p = .839; [tHb]: F_(4.40, 140.92)_ = 2.30, trend p = .055). A significant location×time×group interaction indicated lateralization effects, which were not consistent across time and groups ([O_2_Hb]: F_(2.64, 84.46)_ = 5.00, p = .004; [HHb]: F_(2.49, 79.52)_ = 0.96, p = .404; [tHb]: F_(2.47, 78.98)_ = 4.51, p = .009). Further testing at group level revealed however no significant lateralization effects in the older adults (all LIs <±12), indicating bilateral activation during 0-back, 1-back and 2-back performance. In the young adults, however, a trend was found for the location×time×load interaction, but only for changes of [O_2_Hb] (F_(10, 160)_ = 1.80, p = .065). The location×time interaction was significant ([O_2_Hb]: F_(2.82, 45.15)_ = 5.15, p = .004; [HHb]: F_(2.17, 34.75)_ = 0.96, p = .295; [tHb]: F_(2.68, 42.89)_ = 4.84, p = .007). LIs suggested slight right-hemispheric dominance during 0-back ([O_2_Hb]: LI = −21.7; [HHb]: LI = −4.8; [tHb]: LI = −10.1) and 1-back ([O_2_Hb]: LI = −14.9; [HHb]: LI = −14.5; [tHb]: LI = −19.8), but not during 2-back performance ([O_2_Hb]: LI = −4.6; [HHb]: LI = −0.28; [tHb]: LI = −15.8). Therefore, to establish effects of working-memory load and age, data from the left and right fNIRS channel were analyzed separately.

#### Working-memory load

In the young adults, left prefrontal activation was, overall, larger with increasing working-memory load ([O_2_Hb]: F_(2, 32)_ = 3.98, p = .029; [HHb]: F_(2, 32)_ = 5.99, p = .006; [tHb]: F_(2, 32)_ = 5.48, p = .009). The 2-back vs. 1-back comparison showed increased [O_2_Hb] (p = .007), decreased [HHb] (p = .015), and increased [tHb] (p = .020). The 2-back vs. 0-back comparison revealed increased [O_2_Hb] (trend; p = .058) and increased [tHb] (p = .003). The 1-back vs. 0-back comparison showed decreased [HHb] (p = .005). For the right prefrontal cortex, there were no clear effects of working-memory load in the young adults. Only the 2-back vs. 0-back comparison showed increased [O_2_Hb] (trend; p = .057) and increased [tHb] (p = .016).

In the older adults, both left and right prefrontal activation increased with rising load (left: [O_2_Hb]: F_(2, 32)_ = 10.94, p<.001; [HHb]: F_(2, 32)_ = 2.41, p = .106; [tHb]: F_(2, 32)_ = 11.94, p<.001; right: [O_2_Hb]: F_(2, 32)_ = 10.96, p<.001; [HHb]: F_(2, 32)_ = 7.17, p = .003; [tHb]: F_(2, 32)_ = 6.89, p = .003). The 2-back vs. 1-back comparison revealed increased [O_2_Hb] (left and right: p<.001), decreased [HHb] (left: p = .013; right: p<.001), and increased [tHb] (left: p<.001; right: p = .006) in both hemispheres. The 2-back vs. 0-back comparison revealed increased [O_2_Hb] (left: p = .008; right: p = .003) and increased [tHb] (left and right: p = .001) in both hemispheres. No significant changes of the fNIRS parameters were found for the 1-back vs. 0-back comparison.

#### Group differences

For the left fNIRS channel, significant time×group interactions were found for the 1-back and 2-back task, but not for the 0-back task, suggesting differences in left prefrontal activation over time between young and old. The 1-back condition elicited a significantly different response between groups. Young adults showed a small initial drop of [O_2_Hb] and [tHb], and subsequently an increase of [O_2_Hb] and [tHb]. Contrary to that, older adults showed a decrease of [O_2_Hb] and [tHb] ([O_2_Hb]: F_(3.35, 107.14)_ = 2.97, p = .030; [HHb]: F_(3.00, 95.90)_ = 0.42, p = .741; [tHb]: F_(2.70, 86.26)_ = 3.26, p = .030). During 2-back performance, both groups showed an increase of [O_2_Hb] and a decrease of [HHb], although the time courses significantly differed ([O_2_Hb]: F_(2.43, 77.84)_ = 4.00, p = .016; [HHb]: F_(2.45, 78.35)_ = 4.34, p = .011; [tHb]: F_(2.24, 71.65)_ = 1.83, p = .163).

For the right fNIRS channel, we found significant time×group interaction effects that were similar to the left fNIRS channel for the 1-back and 2-back task (1-back: [O_2_Hb]: F_(2.87, 91.79)_ = 2.93, p = .040; [HHb]: F_(2.68, 85.72)_ = 0.99, p = .393; [tHb]: F_(2.44, 78.15)_ = 2.60, p = .069); 2-back: ([O_2_Hb]: F_(2.46, 78.63)_ = 9.34, p<.001; [HHb]: F_(2.75, 88.09)_ = 4.70, p = .005; [tHb]: F_(2.26, 72.35)_ = 7.59, p = .001).

In summary, no group difference in temporal pattern of prefrontal activation was found for the 0-back task. Older adults showed a decrease of the hemodynamic response during 1-back performance, while young adults showed an increased response. During 2-back performance, young adults showed in comparison to older adults stronger activation of the prefrontal cortex over time.

#### Time course

The time courses of the hemodynamic changes differed between young and older adults. In young adults the changes of [O_2_Hb] and [HHb] during performance of all n-back tasks and the changes of [tHb] during performance of the 2-back task followed a linear trend (p<.05). In the older adults the changes of [O_2_Hb] (left: p = .055; right: p = .048) and [tHb] (left: p = .031; right: p = .078) tended to follow a quadratic course during the 1-back task. For the 2-back task, clear quadratic trends were found ([O_2_Hb]: left and right: p = .002; [HHb]: left: p = .180; right: p = .099; [tHb]: left and right: p = .001).

In addition to this difference in linear vs. quadratic shape of the time-activation curve, we further analyzed group differences in prefrontal activation during the six time segments of the 2-back task. Young adults showed a further increase of [O_2_Hb] and [tHb] and a decrease of [HHb] after the fourth time segment (p≤.01, with the exception of [tHb] left: p = .128). Contrary to that, in older adults the changes of the fNIRS parameters reached a maximum and plateau level during the third time segment. [tHb] significantly declined after the fifth time segment (p<.05).

### Blood pressure

Mean blood pressure slightly increased during performance of the 0-back (young: 0.9±2.0 mmHg; old: 3.2±3.9 mmHg), 1-back task (young: 1.3±2.3 mmHg; old: 4.2±5.8 mmHg), and 2-back task (young: 3.9±4.5 mmHg; old: 6.1±4.9 mmHg) in comparison to baseline measurements. These increases in mean blood pressure did however not significantly differ between groups (0-back: F_(1, 27)_ = 3.85, p = .060; 1-back: F_(1, 27)_ = 3.33, p = .079; 2-back: F_(1, 27)_ = 1.59, p = .218). Therefore, we conclude that the cerebral hemodynamic differences between the groups cannot be explained by systemic changes, but are truly the result of aging effects.

## Discussion

The present study investigated the effects of aging on activation of the prefrontal cortex, by measuring changes of [O_2_Hb], [HHb], and [tHb] during verbal working-memory performance. Based on our results it can be hypothesized that young adults were better able to keep the prefrontal cortex recruited over time during high working-memory load performance. Older adults appear to recruit both hemispheres already at low levels of working-memory load, possibly in an attempt to compensate for the observed aging-related decline in performance.

The fNIRS analyses revealed that in both young and older adults prefrontal activation increased with rising working-memory load. The results of the young adults are in agreement with other fNIRS studies on verbal n-back performance. Hoshi et al. [48], Herrmann et al. [49], and Honma et al. [50] reported that the degree of increase of [O_2_Hb] and decrease of [HHb] showed a positive correlation with working-memory load in young adults. Previous fNIRS studies on cognitive aging suggest aging-related decline in prefrontal activity during cognitive performance. However, to our knowledge, no fNIRS studies using a verbal n-back task in older adults have been published to date. Some fNIRS studies indicate reduced prefrontal lateralization in older adults [29], [30]. Unfortunately, not all previous fNIRS studies performed measurements on both hemispheres or performed analyses on lateralization effects or reported on these. The results of fMRI and EEG studies on the relationship between working-memory load and prefrontal activation are mixed. However, a common finding is that older adults show maximum prefrontal activation at low working-memory loads and that young adults show increasing prefrontal activation up to high working-memory loads [11]–[14]. In our study, the largest prefrontal activity was found during 2-back performance for both the young and the older adults.

Our behavioral results showed that low and high working-memory load resulted in a declined accuracy in comparison to the control condition in older adults, while young adults demonstrated the same level of accuracy in all conditions. We consider it unlikely that the aging-related differences in prefrontal activation are related to task difficulty in general. Barch et al. [38] found that the dorsolateral prefrontal cortex is specifically involved in working- memory function, and that this area is not responsive to task difficulty. Moreover, in the present study, accuracy during 2-back performance was on average very high in both groups. This suggests that working-memory capacity was not exceeded in either of the groups. Furthermore, we found a consistent difference in reaction time between the young adults and elderly, which is in line with the notion that many aspects of information processing become less efficient with increasing age [33]. In both groups reaction times slowed with increasing load. Taken together, our results provide a reliable estimate of the relationship between working-memory load and activation of the prefrontal cortex.

The pattern of prefrontal activation differed between the age groups. Older adults showed bilateral activity during all conditions, whereas young adults showed slight right-hemispheric dominance during 0-back and 1-back performance. These results are in agreement with the HAROLD model ([34], p. 85) which states that “under similar circumstances, prefrontal activity during cognitive performance tends to be less lateralized in older adults than in younger adults”. The function of age-related reductions in asymmetry is unclear, but different interpretations have been introduced such as the dedifferentiation [8] and compensation hypotheses [9]. Our results are in line with CRUNCH [9]. According to CRUNCH, activity in cortical regions is upregulated as task load increases, independent of age. Older adults however, may need to recruit more cortical regions at lower levels of task demand in order to compensate for reduced neural efficiency. In the current study we found that older adults showed bilateral activity already at lower loads. Young adults showed slight right-hemispheric dominance at lower loads. At high task load, young adults no longer showed a lateralization effect, indicating additional recruitment of the left hemisphere.

Furthermore, the time-activation curve during high working-memory load performance differed between groups; it tended to follow a linear course in young adults and a quadratic course in older adults. Maximum and plateau level of activation were reached earlier in older adults than in young adults. Possibly, older adults reached the limit of available neural resources before the young adults did. Alternatively, performance may have become more automatic in older adults. Evidence exists that prefrontal activity increases during initial learning and decreases as a task becomes more practiced [51]. However, we consider this explanation less plausible, because an automatic response would also have occurred in young adults and especially in high performers.

The time course of hemoglobin changes during prolonged cognitive activation has been assessed by means of fNIRS in children [52], in young adults [53]–[55], in middle aged adults [24], and in Alzheimer patients versus older adults [56]. However, we are not aware of any other fNIRS study that directly compared young and older adults during prolonged cognitive activation using the same task.

Butti et al. [57] provided a detailed description of the time course of the fNIRS parameters in the prefrontal cortex of young healthy adults, while they performed a continuous performance task for ten minutes. The task elicited a significant change of the fNIRS parameters which did not remain constant during the task period. The authors concluded that this fluctuation of fNIRS parameters, which was not correlated with behavioral performance, is the result of a complex mechanism of coupling and uncoupling between cerebral blood flow and cerebral metabolic rate of oxygen. In addition, our study indicates that aging has an effect on the time course of [O_2_Hb] and [HHb] changes during sustained activation which is possibly the result of changes in neurovascular coupling that accompany aging, although we could not establish the relationship between fNIRS parameter change and behavioral performance. Neurovascular coupling refers to the relationship between local neural activity and subsequent changes in hemodynamic properties of the surrounding vasculature, including cerebral blood flow, cerebral blood volume, and cerebral metabolic rate of oxygen. The mechanisms that underlie neurovascular coupling are not fully understood. However, evidence exists that these mechanisms alter with aging [58]. Aging is accompanied by a degeneration of the vascular system, probably beginning as early as the fourth decade of life [59]. Regional cerebral blood flow, for example, decreases with age and vessel stiffness is enhanced [60]. These phenomena are accompanied by a reduction in the cerebral metabolic rate of oxygen [61]. Accordingly, fNIRS may be a valuable complementary method to fMRI in unraveling the effects of aging on the time course of the hemodynamic response.

For data analysis it is important to keep the findings of León-Carrión et al. [62], [63] and Butti et al. [57] in mind. Their fNIRS studies showed that exposure to stimuli can cause prefrontal activation lasting long after stimuli cessation and may even induce more robust prefrontal activation after than during the task period. In several fNIRS studies, the mean of a pre- and post-task baseline was calculated to exclude slow drift during the task period by means of a linear fitting procedure. An overshoot of [O_2_Hb] during the post-task period might lead to an overestimation of drift, resulting in an underestimation of the genuine effects. In our study, we also found an substantial overshoot of [O_2_Hb] in both young and older adults after task cessation (data not reported). Therefore, we argue for an analysis procedure that does not incorporate post-task baseline activation.

Finally, we recognize some limitations of our study. Since we only monitored activation of the prefrontal cortex, we cannot rule out the possibility that other brain regions were under- or overactivated in older adults in comparison to young adults. We cannot determine whether bilateral brain activity leads to improved performance or whether an underlying mechanism causes both functional brain reorganization and the decrement in performance. Further, in our study, we found that effects of [O_2_Hb] were not always accompanied by effects of [HHb]. This is in line with the notion that in fNIRS research [O_2_Hb] may be a more robust indicator for changes in regional cerebral blood flow, due to larger changes in amplitude. Since [HHb] changes are less pronounced, this may have led to slightly weaker statistical results.

Future fNIRS studies on cognitive aging should consider the relationship between performance level and prefrontal activation patterns during working-memory performance. For example, groups should be divided into low and high performers or accuracy can be correlated with prefrontal signal change. Recently, this relationship has been considered in fMRI studies [12], [13], [15], [64]. In both young and older adults, differences in prefrontal activation patterns between high and low performers were found. It is suggested that in comparison to low performers, high performers show increased prefrontal activity under high working-memory demands. This might be true for both young and older adults.

To conclude, in this study we examined the effects of aging on prefrontal activation during working-memory performance. The results suggest that young adults are better able to keep the prefrontal cortex recruited over time. Older adults may, already at low levels of working-memory load, recruit both hemispheres possibly in an attempt to compensate for the observed aging-related decline in performance. Also, our study indicates that effects of aging on the time course of hemodynamic processes must be taken into account in the interpretation of neuroimaging studies that rely on blood oxygen levels, such as fMRI.

## References

[1] Salthouse TA, Babcock RL (1991) Decomposing adults age differences in working memory. Dev Psychol 27: 763–776.

[2] Baddeley AD (1992) Working memory. Science 255: 556–559. 1736359 10.1126/science.1736359

[3] Park DC, Lautenschlager G, Hedden T, Davidson NS, Smith AD, et al (2002) Models of visuospatial and verbal memory across the adult life span. Psychol Aging 17: 299–320. 12061414

[4] Grady CL, Craik FIM (2000) Changes in memory processing with age. Curr Opin Neurobiol 10: 224–231. 10753795 10.1016/s0959-4388(00)00073-8

[5] Reuter-Lorenz PA, Jonides J, Smith EE, Hartley A, Miller A, et al (2000) Age differences in the frontal lateralization of verbal and spatial working memory revealed by PET. J Cogn Neurosci 12: 174–187. 10769314 10.1162/089892900561814

[6] Mitchell KJ, Johnson MK, Raye CL, D'Esposito M (2000) fMRI evidence of age-related hippocampal dysfunction in feature binding in working memory. Cogn Brain Res 10: 197–206. 10.1016/s0926-6410(00)00029-x 10978709

[7] Cabeza R, Daselaar SM, Dolcos F, Prince SE, Budde M, et al (2004) Task-independent and task-specific age effects on brain activity during working memory, visual attention and episodic retrieval. Cereb Cortex 14: 364–375. 15028641 10.1093/cercor/bhg133

[8] Li S-C, Lindenberger U, Sikström S (2001) Aging cognition: from neuromodulation to representation. Trend Cogn Sci 5: 479–486. 10.1016/s1364-6613(00)01769-1 11684480

[9] Reuter-Lorenz PA, Cappell KA (2008) Neurocognitive aging and the compensation hypothesis. Curr Dir Psychol Sci 17: 177–182.

[10] Meulenbroek O, Kessels RPC, de Rover M, Petersson KM, Olde Rikkert MGM, et al (2010) Age-effects on associative object-location memory. Brain Res 1315: 100–110. 20018180 10.1016/j.brainres.2009.12.011

[11] Mattay VS, Fera F, Tessitore A, Hariri AR, Berman KF, et al (2006) Neurophysiological correlates of age-related changes in working memory capacity. Neurosci Lett 392: 32–37. 16213083 10.1016/j.neulet.2005.09.025

[12] Nyberg L, Dahlin E, Stigsdotter Neely A, Bäckman L (2009) Neural correlates of variable working memory load across adult age and skill: dissociative patterns within the fronto-parietal network. Scand J Psychol 50: 41–46. 18705668 10.1111/j.1467-9450.2008.00678.x

[13] Nagel IE, Preuschhof C, Li S-C, Nyberg L, Bäckman L, et al (2011) Load modulation of the BOLD response and connectivity predicts working memory performance in younger and older adults. J Cogn Neurosci 23: 2030–2045. 20828302 10.1162/jocn.2010.21560

[14] Missonnier P, Herrmann FR, Rodriguez C, Deiber M-P, Millet P, et al (2011) Age-related differences on event-related potentials and brain rhythm oscillations during working memory activation. J Neural Transm 118: 945–955. 21331458 10.1007/s00702-011-0600-2

[15] Schneider-Garces NJ, Gordon BA, Brumback-Peltz CR, Shin E, Lee Y, et al (2009) Span, CRUNCH, and beyond: working memory capacity and the aging brain. J Cogn Neurosci 22: 655–669. 10.1162/jocn.2009.21230 PMC3666347 19320550

[16] Villringer A, Chance B (1997) Non-invasive optical spectroscopy and imaging of human brain function. Trends Neurosci 20: 435–442. 9347608 10.1016/s0166-2236(97)01132-6

[17] Hoshi Y (2003a) Functional near-infrared optical imaging: utility and limitations in human brain mapping. Psychophysiology 40: 511–520. 14570159 10.1111/1469-8986.00053

[18] Obrig H, Villringer A (2003) Beyond the visible – imaging the human brain with light. J Cereb Blood Flow Metab 23: 1–18. 10.1097/01.WCB.0000043472.45775.29 12500086

[19] Liu H, Boas DA, Zhang Y, Yodh AG, Chance B (1995a) Determination of optical properties and blood oxygenation in tissue using continuous NIR light. Phys Med Biol 40: 1983–1993. 8587945 10.1088/0031-9155/40/11/015

[20] Liu H, Chance B, Hielscher AH, Jacques SL, Tittel FK (1995b) Influence of blood vessels on the measurement of hemoglobin oxygenation as determined by time resolved reflectance spectroscopy. Med Phys 22: 1209–1217. 7476706 10.1118/1.597520

[21] Logothetis NK, Wandell BA (2004) Interpreting the BOLD signal. Annu Rev Physiol 66: 735–769. 14977420 10.1146/annurev.physiol.66.082602.092845

[22] Steinbrink J, Villringer A, Kempf F, Haux D, Boden S, et al (2006) Illuminating the BOLD signal: combined fMRI-fNIRS studies. Magn Reson Imaging 24: 495–505. 16677956 10.1016/j.mri.2005.12.034

[23] Fazli S, Mehnert J, Steinbrink J, Curio G, Villringer A, et al (2012) Enhanced performance by a hybrid NIRS-EEG brain computer interface. Neuroimage 59: 519–529. 21840399 10.1016/j.neuroimage.2011.07.084

[24] Hock C, Müller-Spahn F, Schuh-Hofer S, Hofmann M, Dirnagl U, et al (1995) Age dependency of changes in cerebral hemoglobin oxygenation during brain activation: a near-infrared spectroscopy study. J Cereb Blood Flow Metab 15: 1103–1108. 7593343 10.1038/jcbfm.1995.137

[25] Kameyama M, Fukuda M, Uehara T, Mikuni M (2004) Sex and age dependencies of cerebral blood volume changes during cognitive activation: a multichannel near-infrared spectroscopy study. Neuroimage 22: 1715–1721. 15275927 10.1016/j.neuroimage.2004.03.050

[26] Schroeter ML, Zysset S, Kruggel F, von Cramon DY (2003) Age dependency of the hemodynamic response as measured by functional near-infrared spectroscopy. Neuroimage 19: 555–564. 12880787 10.1016/s1053-8119(03)00155-1

[27] Kwee IL, Nakada T (2003) Dorsolateral prefrontal lobe activation declines significantly with age: functional NIRS study. J Neurol 250: 525–529. 12736729 10.1007/s00415-003-1028-x

[28] Holtzer R, Mahoney JR, Izzetoglu M, Izzetoglu K, Onaral B, et al (2011) fNIRS study of walking and walking while talking in young and old individuals. J Gerontol A Biol Sci Med Sci 66: 879–887. 21593013 10.1093/gerona/glr068 PMC3148759

[29] Herrmann MJ, Walter A, Ehlis A-C, Fallgatter AJ (2006) Cerebral oxygenation changes in the prefrontal cortex: effects of age and gender. Neurobiol of Aging 27: 888–894. 10.1016/j.neurobiolaging.2005.04.013 16023767

[30] Tsjuii T, Okada M, Watanabe S (2010) Effects of aging on hemispheric asymmetry in inferior frontal cortex activity during belief-bias syllogistic reasoning: A near-infrared spectroscopy study. Behav Brain Res 210: 178–183. 20171989 10.1016/j.bbr.2010.02.027

[31] Jansma JM, Ramsey NF, Coppola R, Kahn RS (2000) Specific versus nonspecific brain activity in an parametric n-back task. Neuroimage 12: 688–697. 11112400 10.1006/nimg.2000.0645

[32] Owen AM, McMillan KM, Laird AR, Bullmore E (2005) N-back working memory paradigm: a meta-analysis of normative functional neuroimaging studies. Hum Brain Mapp 25: 46–59. 15846822 10.1002/hbm.20131 PMC6871745

[33] Salthouse TA (1996) The processing-speed theory of adult age differences in cognition. Psychol Rev 103: 403–428. 8759042 10.1037/0033-295x.103.3.403

[34] Cabeza R (2002) Hemispheric asymmetry reduction in older adults: the HAROLD model. Psychol Aging 17: 85–100. 11931290 10.1037//0882-7974.17.1.85

[35] Folstein MF, Folstein SE, McHugh PR (1975) “Mini-mental state”: A practical method for grading the cognitive state of patients for the clinician. J Psychiatr Res 12: 189–198. 1202204 10.1016/0022-3956(75)90026-6

[36] Verhage F (1964) Intelligentie en leeftijd bij volwassenen en bejaarden. Van Gorcum, Assen

[37] Schmand B, Lindeboom J, van Harskamp F (1992) De Nederlandse leestest voor volwassen. Swets & Zeitlinger, Lisse

[38] Barch DM, Braver TS, Nystrom LE, Forman SD, Noll DC, et al (1997) Dissociating working memory from task difficulty in human prefrontal cortex. Neuropsychologia 35: 1373–1380. 9347483 10.1016/s0028-3932(97)00072-9

[39] Sakatani K, Yamashita D, Yamanaka T, Oda M, Yamashita Y, et al (2006) Changes of cerebral blood oxygenation and optical pathlength during activation and deactivation in the prefrontal cortex measured by time-resolved near infrared spectroscopy. Life Sci 78: 2734–2741. 16360709 10.1016/j.lfs.2005.10.045

[40] van de Ven MJT, Colier WNJM, van der Sluijs MC, Walraven D, Oeseburg B, et al (2001) Can cerebral blood volume be measured reproducibly with an improved near infrared spectroscopy system? J Cereb Blood Flow Metab 21: 110–113. 11176276 10.1097/00004647-200102000-00002

[41] van Beek AHEA, Lagro J, Olde Rikkert MGM, Zhang R, Claassen JAHR (2011) Oscillations in cerebral blood flow and cortical oxygenation in Alzheimers's disease. Neurobiol Aging 33: 428.e21–428.e31. 21208686 10.1016/j.neurobiolaging.2010.11.016

[42] Haeussinger FB, Heinzel S, Hahn T, Schecklmann M, Ehlis A-C, et al (2011) Simulation of near-infrared light absorption considering individual head and prefrontal cortex anatomy: implications for optical neuroimaging. PLoS ONE 6: e26377. 22039475 10.1371/journal.pone.0026377 PMC3200329

[43] Fukui Y, Ajichi Y, Okada E (2003) Monte Carlo prediction of near-infrared light propagation in realistic adults and neonatal head models. Appl Optics 42: 2881–2887. 10.1364/ao.42.002881 12790436

[44] Okamoto M, Dan H, Sakamoto K, Takeo K, Shimizu K, et al (2004) Three-dimensional probalistic anatomical cranio-cerebral correlation via the international 10–20 system oriented for transcranial functional brain mapping. Neuroimage 21: 99–111. 14741647 10.1016/j.neuroimage.2003.08.026

[45] Duncan A, Meek JH, Clemence M, Elwell CE, Fallon P, et al (1996) Measurement of cranial optical path length as a function of age using phase resolved near infrared spectroscopy. Pediatr Res 39: 889–894. 8726247 10.1203/00006450-199605000-00025

[46] Claassen JAHR, Colier WNJM, Jansen RWMM (2006) Reproducibility of cerebral blood volume measurements by near infrared spectroscopy in 16 healthy elderly subjects. Physiol Meas 27: 255–264. 16462012 10.1088/0967-3334/27/3/004

[47] Grier JB (1971) Nonparametric indexes for sensitivity and bias: computing formulas. Psychol Bull 75: 424–429. 5580548 10.1037/h0031246

[48] Hoshi Y, Tsou BH, Billock VA, Tanosaki M, Iguchi Y, et al (2003b) Spatiotemporal characteristics of hemodynamic changes in the human lateral prefrontal cortex during working memory tasks. Neuroimage 20: 1493–1504. 14642462 10.1016/s1053-8119(03)00412-9

[49] Herrmann MJ, Walter A, Schreppel T, Ehlis A-C, Pauli P, et al (2007) D4 receptor gene variation modulates activation of prefrontal cortex during working memory. Eur J Neurosci 26: 2713–2718. 17970718 10.1111/j.1460-9568.2007.05921.x

[50] Honma M, Soshi T, Kim Y, Kuriyama K (2010) Right prefrontal activity reflects the ability to overcome sleepiness during working memory tasks: a functional near-infrared spectroscopy. PLoS ONE 5: e12923. 20886073 10.1371/journal.pone.0012923 PMC2944865

[51] Petersen SE, van Mier H, Fiez JA, Raichle ME (1998) The effects of practice on the functional anatomy of task performance. Proc Natl Acad Sci U S A 95: 853–860. 9448251 10.1073/pnas.95.3.853 PMC33808

[52] Matsuda G, Hiraki K (2006) Sustained decrease in oxygenated hemoglobin during video games in the dorsal prefrontal cortex: a NIRS study of children. Neuroimage 29: 706–711. 16230030 10.1016/j.neuroimage.2005.08.019

[53] Heekeren HR, Obrig H, Wenzel R, Eberle K, Ruben J, et al (1997) Cerebral haemoglobin oxygenation during sustained visual stimulation - a near-infrared spectroscopy study. Phil Trans R Soc B 352: 743–750. 9232863 10.1098/rstb.1997.0057 PMC1691960

[54] Fallgatter AJ, Strik WK (1997) Right frontal activation during the continuous performance test assessed with near-infrared spectroscopy in healthy subjects. Neurosci Lett 223: 89–92. 9089680 10.1016/s0304-3940(97)13416-4

[55] Fallgatter AJ, Strik WK (1998) Frontal brain activation during the Wisconsin Card Sorting Test assessed with two-channel near-infrared spectroscopy. Eur Arch Psychiatry Clin Neurosci 248: 245–249. 9840371 10.1007/s004060050045

[56] Hock C, Villringer K, Müller-Spahn F, Wenzel R, Heekeren H, et al (1997) Decrease in parietal cerebral hemoglobin oxygenation during performance of a verbal fluency task in patients with Alzheimer's disease monitored by means of near-infrared spectroscopy (NIRS) -correlation with simultaneous rCBF-PET measurements. Brain Res 755: 293–303. 9175896 10.1016/s0006-8993(97)00122-4

[57] Butti M, Contini D, Molteni E, Caffini M, Spinelli L, et al (2009) Effect of prolonged stimulation on cerebral hemodynamic: a time-resolved fNIRS study. Med Phys 36: 4103–4114. 19810483 10.1118/1.3190557

[58] D'Esposito M, Deouell LY, Gazzaley A (2003) Alterations in the BOLD fMRI signal with ageing and disease: a challenge for neuroimaging. Nature Rev Neurosci 4: 863–872. 14595398 10.1038/nrn1246

[59] Farkas E, Luiten PGM (2001) Cerebral microvascular pathology in aging and Alzheimer's disease. Prog Neurobiol 64: 575–611. 11311463 10.1016/s0301-0082(00)00068-x

[60] Marin J (1995) Age-related changes in vascular responses: a review. Mech Ageing Dev 79: 71–114. 7616768 10.1016/0047-6374(94)01551-v

[61] Marchal G, Rioux P, Petit-Taboué MC, Sette G, Travère JM, et al (1992) Regional cerebral oxygen consumption, blood flow, and blood volume in healthy human aging. Arch Neurol 49: 1013–1020. 1417508 10.1001/archneur.1992.00530340029014

[62] León-Carrión J, Damas J, Izzetoglu K, Pourrezai K, Martín-Rodríguez JF, et al (2006) Differential time course and intensity of PFC activation for men and women in response to emotional stimuli: a functional near-infrared spectroscopy (fNIRS) study. Neurosci Lett 403: 90–95. 16716510 10.1016/j.neulet.2006.04.050

[63] León-Carrión J, Martín-Rodríguez JF, Damas-López J, Pourrezai K, Izzetoglu K, et al (2007) A lasting post-stimulus activation on dorsolateral prefrontal cortex is produced when processing valence and arousal in visual affective stimuli. Neurosci Lett 422: 147–152. 17601668 10.1016/j.neulet.2007.04.087

[64] Nagel IE, Preuschhof C, Li S-C, Nyberg L, Bäckman L, et al (2009) Performance level modulates adult age differences in brain activation during spatial working memory. Proc Natl Acad Sci U S A 106: 22552–22557. 20018709 10.1073/pnas.0908238106 PMC2799744

